# Research on Impact-Induced Reaction Characteristics of Al_2_Ce/AP Reactive Material

**DOI:** 10.3390/nano16080463

**Published:** 2026-04-14

**Authors:** Shoujia Li, Beichen Zhang, Lin Peng, Yan Liu, Hongwei Zhao, Xiaoxia Lu, Pengyu Bi

**Affiliations:** Chemical Defense Institute, Academy of Military Sciences, Beijing 102205, China; lee19950222@163.com (S.L.); zbc992004683@126.com (B.Z.); pl981210@163.com (L.P.); liuyanwbl@163.com (Y.L.); zhw211@126.com (H.Z.)

**Keywords:** aluminum–cerium alloy, ammonium perchlorate, mechanical properties, thermal decomposition, impact ignition

## Abstract

To overcome the low strength of conventional polytetrafluoroethylene/aluminum (PTFE/Al) reactive materials and the insufficient reaction efficiency of aluminum, this study introduces highly reactive aluminum–cerium alloys (Al-Ce-1#, -2#, and -3#, with Ce contents of 30, 50, and 70%, respectively; the primary phase in Al-Ce-3# is Al_2_Ce) with a multiscale structural design (comprising both micron-sized and nano-sized particles) into an ammonium perchlorate (AP) matrix. Al/AP reactive materials and Al-Ce/AP reactive materials with varying Ce contents were prepared. The thermal decomposition characteristics, dynamic mechanical properties, and impact ignition behavior were systematically investigated using differential scanning calorimetry (DSC) and split Hopkinson pressure bar (SHPB) experiments. The results demonstrate that the addition of Al_2_Ce significantly alters the thermal decomposition process of AP, substantially lowering its decomposition temperature (by approximately 69 °C) and promoting concentrated exothermic decomposition. SHPB tests reveal that Al_2_Ce/AP composites exhibit higher dynamic yield strength and flow stress than the Al/AP, accumulating faster strain energy density under impact loading, which indicates a more violent fragmentation failure mode. This enhanced mechanical failure behavior, which provides highly reactive interfaces and promotes hotspot formation, synergizes with the catalytic effect of Al_2_Ce on AP decomposition. Together, these mechanisms jointly improve the impact ignition sensitivity of the material, significantly lowering its ignition threshold and shortening its combustion duration. This study confirms that Al_2_Ce/AP is a novel reactive material combining excellent dynamic mechanical properties with outstanding impact reactivity, providing theoretical and technical support for the application of highly reactive rare-earth aluminum alloys in aluminum-based reactive materials.

## 1. Introduction

Reactive materials are a class of materials made by combining two or more non-explosive solids, and they usually remain inert under normal conditions. When they collide with an intended target or are subjected to sufficiently strong mechanical stimuli, a reaction is triggered, rapidly releasing heat and energy [1,2,3]. Typically, reactive materials consist of metallic fuels, metal oxides, oxidizers, metastable intermolecular composites (MICs), and matrix materials [3,4,5].

Aluminum (Al) has been widely used in reactive materials due to its high energy density (31.0 MJ/kg) and economic advantages, such as in Bi_2_O_3_/Al [6], CuO/Al [7], and Fe_2_O_3_/Al [8] systems. Fluoropolymers, such as polytetrafluoroethylene (PTFE), can serve as oxidizers. Some researchers have also proposed that certain oxide matrices can use inorganic oxidizers, such as ammonium perchlorate (AP) and potassium perchlorate (KP) [9]. Currently, PTFE/Al (PA) materials are the most used reactive materials. Ge et al. [10], through split Hopkinson pressure bar (SHPB) dynamic loading combined with high-speed photography and strain measurement, obtained the strain rate and stress thresholds for the ignition reaction of PTFE/Al reactive materials and demonstrated that material deformation and failure are important factors inducing ignition. Ren et al. [11] designed PTFE/Al reactive materials with different initial defects and studied the influence of defects on the ignition behavior. The results showed that the material’s ignition threshold significantly decreased with increasing initial defects. Geng et al. [12] studied PTFE/Al reactive materials with inclined structures. The results indicated that the material’s mechanical response depends on its shape, and samples with different inclined structures experienced different dynamic compression–shear states, leading to different ignition behaviors. However, the low strength of fluoropolymer matrices still poses certain limitations for their application in engineering fields [13,14]. On the other hand, aluminum has a slow reaction rate, and metallic aluminum tends to condense into large, unreacted aluminum spheres during combustion, reducing the effective utilization efficiency of aluminum and severely hindering the energy release of the reactive material system [15,16].

Rare-earth cerium (Ce) can regulate or catalyze oxidation/reduction reaction processes and is therefore widely used in manufacturing and catalytic energy fields [17,18,19]. In the field of energetic damage, scientists first introduced rare-earth elements into incendiary systems as early as the 1950s [20]. Ce is the most abundant rare-earth element on Earth, with reserves accounting for about 0.0046% of the Earth’s crust by weight, and its crustal abundance is higher than that of copper (Cu) [21]. Its surface appears iron-gray, with a specific gravity of 6.77 g/cm^3^ and a melting point of 799 °C. Ce reacts readily at room temperature, especially in humid air [22]. The atomic number of Ce is 58, with an electron configuration of [Xe] 4f^1^ 5d^1^ 6s^2^. It has two oxidation states, Ce(III) ([Xe] 4f^1^) and Ce(IV) ([Xe]), with Ce(IV) cerium dioxide (CeO_2_) being its primary oxide form [23,24]. Currently, rare-earth metals like Ce used in incendiaries typically exist in the form of alloys [25,26] or surface passivation [27,28] to ensure their internal compatibility and storage stability meet usage requirements. Sharma et al. [29] prepared cerium dioxide (CeO_2_) nanoparticles (NPs) via a green synthesis method. Introducing CeO_2_ NPs into AP significantly reduced the decomposition temperature of AP by 130 °C and lowered the activation energy, indicating excellent thermal catalytic performance of CeO_2_ NPs. Wang et al. [30] studied the effect of cerium trifluoride (CeF_3_) on the combustion performance of nano-aluminum powder (n-Al). The results showed that CeF_3_ shortened the combustion time of n-Al, but increasing CeF_3_ content suppressed the energy release of the Al.

To gain an in-depth understanding of the ignition performance of Ce-containing reactive materials under dynamic loading and the mechanism by which metallic cerium influences the thermodynamic properties of the system during the impact response, this study introduces metallic aluminum and three highly reactive aluminum–cerium alloys with different cerium contents (with mass ratios of Al to Ce are 7:3, 5:5, and 3:7, designated as Al-Ce-1#, Al-Ce-2#, and Al-Ce-3#, respectively; where the main crystal phase of Al-Ce-3# is Al_2_Ce).

Four metals went into the inorganic oxidizer AP, preparing four reactive material systems: Al/AP and three aluminum–cerium alloy/AP systems. The dynamic mechanical properties and thermal decomposition characteristics of these materials were investigated using the split Hopkinson pressure bar (SHPB) and differential scanning calorimetry (DSC). The mechanical and ignition properties of the different reactive materials were obtained. Furthermore, the influence patterns of Al_2_Ce on the mechanical properties of the AP, as well as the influence patterns of Al and Al_2_Ce on the thermal decomposition performance of the AP, were analyzed. This work provides new insights for the design and application of highly reactive rare-earth materials in Al-based reactive materials.

## 2. Experimental Section

### 2.1. Material Preparation

**Figure 1:**
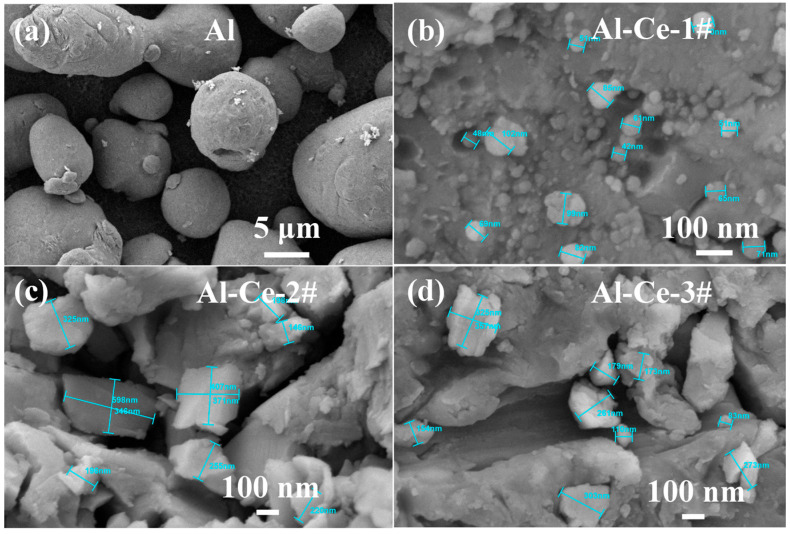
SEM images of metals with different Al mass ratios. (**a**) pure Al (100%Al); (**b**) Al-Ce-1# (70%Al); (**c**) Al-Ce-2# (50%Al); (**d**) Al-Ce-3# (30%Al).

To investigate the effect of different Ce contents on the energy release of the AP system, four formulation systems were designed based on oxygen balance calculations: Al/AP and three aluminum–cerium alloy/AP systems with different mass ratios. The specific formulations are presented in Table 1. The aluminum–cerium alloy powders were prepared via vacuum melting and ball milling in an argon atmosphere, with cerium contents of 30%, 50%, and 70%, designated as Al-Ce-1#, Al-Ce-2#, and Al-Ce-3# (the primary crystalline phase of Al-Ce-3# is Al_2_Ce). For comparison, the SEM image of the as-received atomized pure Al powder is presented in Figure 1d. The Al particles exhibit a predominantly spherical morphology with a smooth surface, in contrast to the irregular, multi-scale structures of the ball-milled Al-Ce alloy powders shown in Figure 1a–c.

Thus, the Al-Ce alloys combine the high strength and reactivity of nanomaterials with the toughness and processing stability of micron-sized materials. The following materials were employed: Atomized aluminum powder, 75~150 μm (Longxi Northwest Aluminum Jiuding Powder Material Co., Ltd., Shanghai, China); ultrafine AP, 10 μm (industrial grade, North Xin’an Chemical Co., Ltd., Taiyuan, China); fluoroelastomer (FKM), grade FKM2603 (industrial grade, Zhonghao Chenguang Chemical Research Institute Co., Ltd., Zigong, China); ethyl acetate, analytical grade (China National Medicines Co., Ltd., Beijing, China).

**Figure 2:**
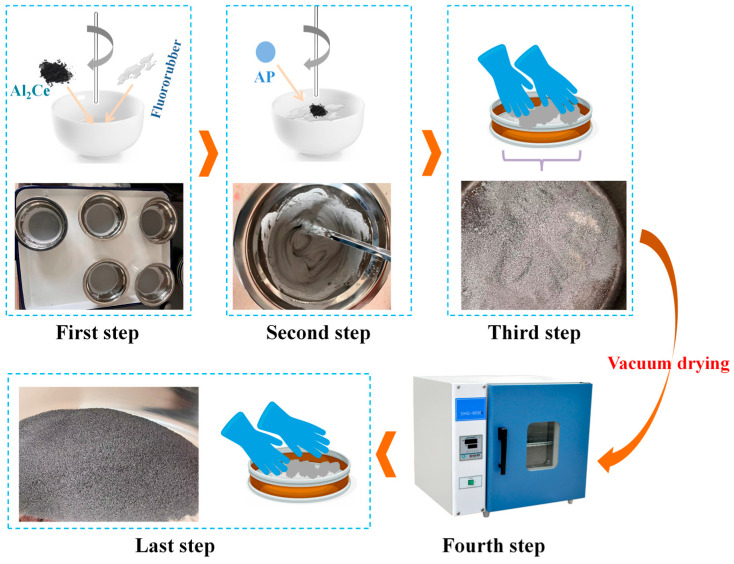
Preparation process of metal/AP composite system.

The preparation and molding process for the reactive metal/AP reactive materials are illustrated in Figure 2. Before the experiment, the FKM binder was dissolved in ethyl acetate and ultrasonically dispersed to prepare a test sample solution with a concentration of 10%. First, the FKM/ethyl acetate solution was added to the reactive metal powder and stirred until homogeneous. Next, the AP was added and the mixture was stirred thoroughly until it reached a semi-dry state due to solvent evaporation. The mixture was then gently tapped through a 40-mesh sieve to remove agglomerated particles, followed by manual homogenization. This sieving and homogenization step was repeated twice. Subsequently, the mixture was transferred to a vacuum drying oven and dried at 60 °C under a pressure of −0.1 kPa for 4 h. The obtained powder mixtures were sealed and stored in a desiccator. Finally, 1.00 g of the sample was accurately weighed, transferred to a cylindrical mold with a diameter of 10 mm, and pressed into pellets under a pressure of 6 MPa with a dwell time of 10 s. The resulting pellets were sealed for storage.

**Figure 3:**
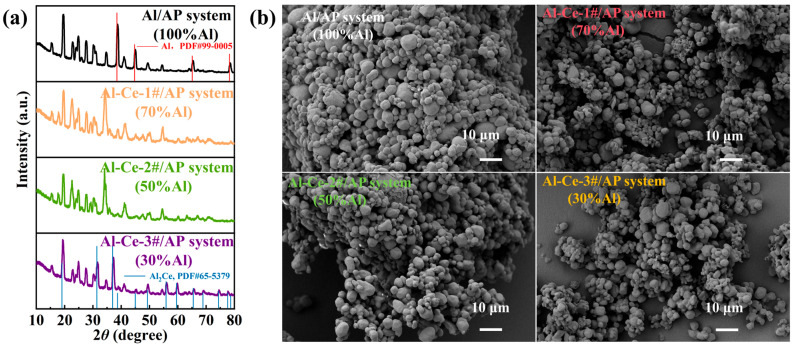
(**a**) XRD patterns and (**b**) SEM images of active metal/AP composite systems.

Figure 3 presents the XRD patterns and SEM micrographs of the different reactive materials. The XRD patterns indicate that the crystalline diffraction peaks of the Al/AP consist primarily of metallic aluminum and AP. The diffraction peaks of Al match the standard pattern (PDF#99-0005). The crystalline diffraction peaks of the Al-Ce-3#/AP system mainly consist of the characteristic peaks of Al_2_Ce and AP, with Al_2_Ce matching the standard card (PDF#65-5379). SEM analysis showed, that after processing, the four reactive materials formed a dense, coated granular structure with no discrete particles observed, thereby eliminating the influence of process errors on subsequent experiments.

### 2.2. Experimental

Thermal Decomposition Test: Thermal decomposition tests were performed using a TG/DTA instrument (TG/DTA7300, EXSTAR, Tokyo, Japan) to study the effect of different reactive metals on the thermal decomposition performance of AP. Approximately 2 mg of sample was weighed for testing using an alumina crucible. The temperature range was from 50 °C to 450 °C at a heating rate of 10 °C/min under an argon atmosphere with a gas flow rate of 40 mL/min.

**Figure 4:**
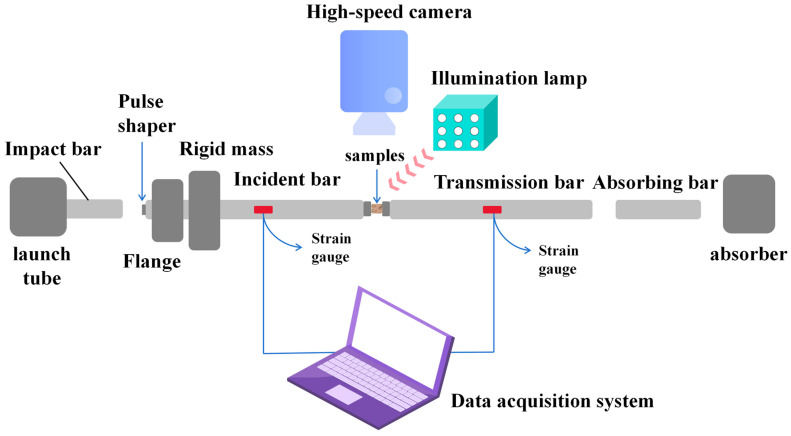
Schematic diagram of a single-loading SHPB experimental system.

Dynamic Response and Ignition Performance Test: During conventional SHPB loading, the reflected tensile wave from the interface between the specimen and the incident bar propagates back to the impact end of the incident bar, where it reflects again as a compressive wave, reloading the specimen. This repeated loading process can occur multiple times, leading to inconsistencies between the actual stress state of the recovered specimen and the obtained stress–strain curve [31]. Nemat-Nasser et al. [32,33] improved the loading end of the incident bar to generate a following tensile wave, thereby ensuring the specimen is subjected to only a single compressive stress pulse. Subsequently, with further research, single-loading device systems have become simpler and more operable [34], as shown in Figure 4. A transmission flange is attached to the impact end of the incident bar, and a fixed mass block is mounted on the test bench. A hole is reserved in the mass block to allow the incident bar to pass through while the flange cannot. When the gap between the flange and the mass block is appropriate, a single pulse loading on the specimen can be reliably achieved.

Before the experiment, a gap of width *h* is left between the flange and the mass block. When the striker bar impacts the incident bar, a compressive pulse is generated. Once the entire pulse has passed and the displacement of the impact end face reaches *h*, the flange and mass block form a fixed-wall constraint. Subsequently, the reflected tensile wave from the interface between the incident bar and the specimen propagates back. When this wave reaches the fixed-wall constraint at the incident bar’s flange, it is still reflected as a tensile wave, and the specimen will not experience secondary loading. Based on one-dimensional stress wave theory and elastic bar collision analysis, when the striker bar and incident bar have the same cross-sectional area and material, the required gap width *h* is given as follows [35]:

(1) $$h=\frac{L_{0}V_{0}}{c_{0}}$$

where *L*_0_ and *V*_0_ are the length and velocity of the striker bar, and *c*_0_ is the material’s sound speed. Furthermore, if a pulse shaper is used during the loading experiment, the formula for calculating the reserved gap width is no longer valid. Under these conditions, the reserved gap width needs to be determined through experimental calibration.

## 3. Results and Discussion

### 3.1. Thermal Decomposition Performance

**Figure 5:**
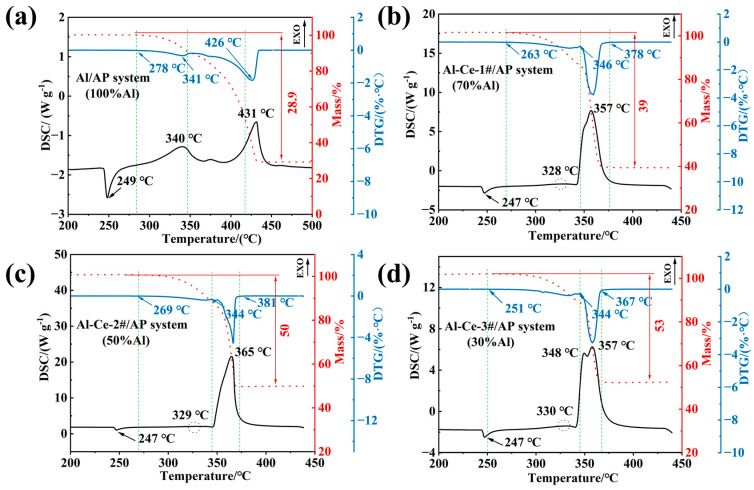
TG-DSC results: (**a**) Al/AP; (**b**) Al-Ce-1#/AP; (**c**) Al-Ce-2#/AP; (**d**) Al-Ce-3#/AP.

To investigate the slow oxidation and decomposition process of different active metal/AP systems under oxygen balanced conditions, and to analyze the influence of metallic Ce and its content on the thermal decomposition performance, DSC tests were conducted on the four materials. Figure 5 shows the TG-DSC curves of the four AP based reactive materials. The thermal decomposition process of the Al/AP system is largely consistent with that of pure AP, proceeding in three distinct stages. The first stage is characterized by an endothermic peak at approximately 249 °C, corresponding to the crystalline phase transition of AP, consistent with the literature [36]. The second stage is the low temperature decomposition of AP. The decomposition initiates at around 278 °C and reaches its maximum rate at approximately 341 °C. This stage involves the decomposition of AP after crystal transition, producing NH_3_ and HClO_4_. As the temperature further increases, the reaction rate slows as a significant portion of the NH_3_ and HClO_4_ becomes adsorbed onto the AP surface. The third stage is the high temperature decomposition of AP, which requires a higher temperature. The gases from the further decomposition of HClO_4_ oxidize NH_3_, ultimately generating gases such as NO and NO_2_ [37], reaching the fastest decomposition rate around 410 °C. As the temperature further increases to 426 °C, the high temperature decomposition of AP concludes.

It is evident that the addition of metallic Al has little effect on the decomposition process of AP. The high temperature decomposition of the Al/AP system occurs at a slightly lower temperature. This shift may be due to the high thermal conductivity of Al, allowing heat required by the system to be promptly transferred to AP particles, thereby promoting their decomposition.

As shown in Figure 5b, the thermal decomposition behavior of the Al-Ce-1#/AP system is markedly different from that of Al/AP. After introducing Al-Ce-1# into the AP system, the low temperature and high temperature decomposition processes of AP merge and occur concentratedly around 350~360 °C, releasing a substantial amount of heat. This is attributed to the high reactivity of Ce, which accelerates the decomposition process, leading to more concentrated energy release. In contrast, after adding Al-Ce-3# with 70%Ce content, the DSC decomposition curve exhibits two distinct peaks at 348 °C and 357 °C. Compared to other systems, decomposition tends to occur at lower temperatures. Furthermore, the measured residual mass of the three Al-Ce/AP systems after decomposition is significantly higher. When combined with the observed heat release, this further indicates that a reaction occurred between the alloy and AP.

**Figure 6:**
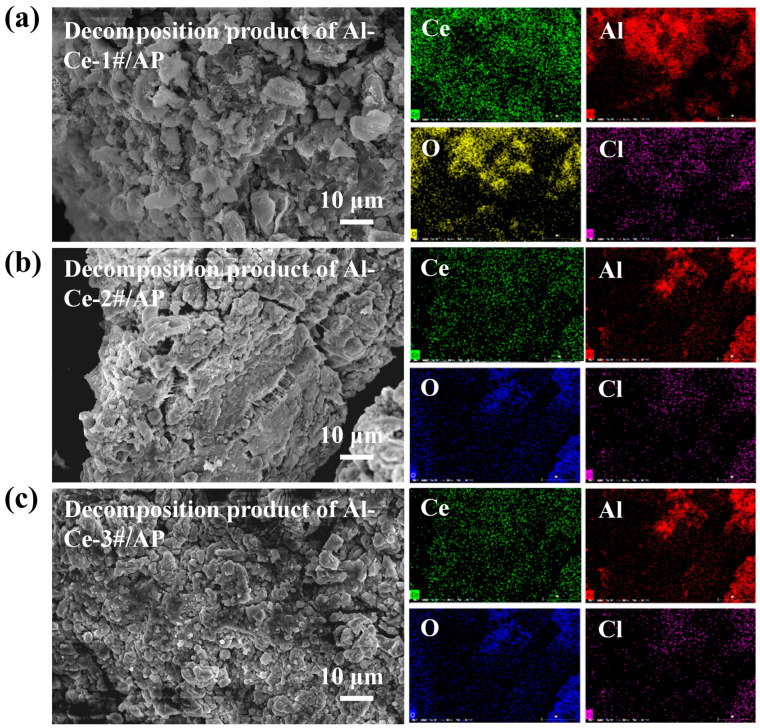
SEM morphology images and EDS elemental surface analysis of thermal decomposition products from the four AP systems: (**a**) Al-Ce-1#/AP; (**b**) Al-Ce-2#/AP; (**c**) Al-Ce-3#/AP.

Figure 6 shows the surface morphology and elemental distribution maps of the thermal decomposition products of the three Al-Ce/AP systems. SEM images reveal that after thermal decomposition, the surfaces of the Al-Ce/AP systems become rough and porous. This altered microstructure is likely more conducive to sample fragmentation under impact loading. EDS results show that Cl element is present in the decomposition products of all three systems, indicating that HClO_4_ produced during the low temperature decomposition of AP undergoes oxidation reactions with the Al-Ce alloy. After a series of reactions, Cl remains in the thermal decomposition products. These results indicate that the introduction of Al_2_Ce directly engages in oxidation reactions with AP. As shown in Table 2, the intense exothermic process significantly lowers the decomposition peak temperature of the system (by up to approximately 69 °C) and makes the decomposition process more concentrated and violent.

### 3.2. Dynamic Mechanical Properties Under SHPB Testing

#### 3.2.1. Ignition Behavior and Mechanical Response Analysis at 19.3 m/s Impact Velocity

**Figure 7:**
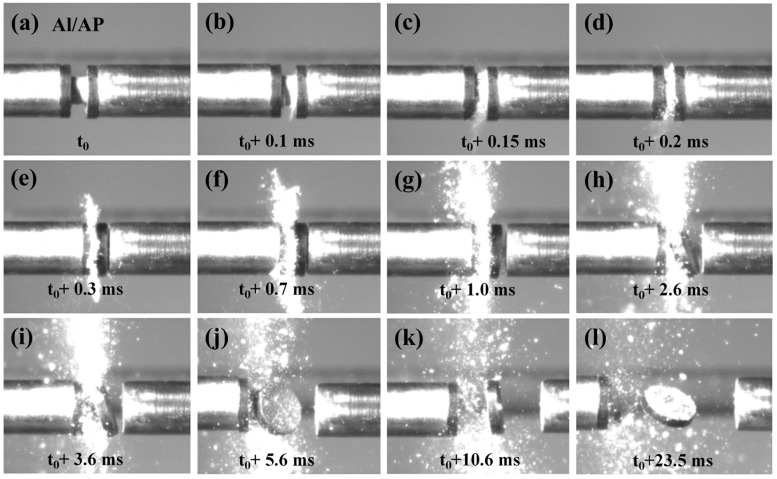
Transient ignition patterns of the Al/AP system under 19.3 m/s impact velocity. (**a**) Specimen before impact; (**b**–**l**) Specimen during impact at various times.

To investigate the influence of Al-Ce alloys with different Ce contents on the ignition performance and dynamic mechanical properties of the Al/AP system, SHPB tests were conducted. A high-speed camera was used to capture the transient ignition morphology of the samples. Figure 7 presents a series of high-speed photographs showing the transient ignition photos of the Al/AP system material at different time points. When the striker bar impacts the incident bar at a velocity of 19.3 m/s, generating a compressive pulse that loads the specimen at 19.3 m/s, the specimen begins to compress and expand laterally at 0.15 ms. At 0.7 ms, after intense impact, the specimen undergoes severe internal fragmentation. Friction between these fragments leads to hot spot accumulation and subsequent ignition. At 3.6 ms, the impact produces the maximum spark intensity, and the entire reaction process lasts approximately 23.5 ms.

**Figure 8:**
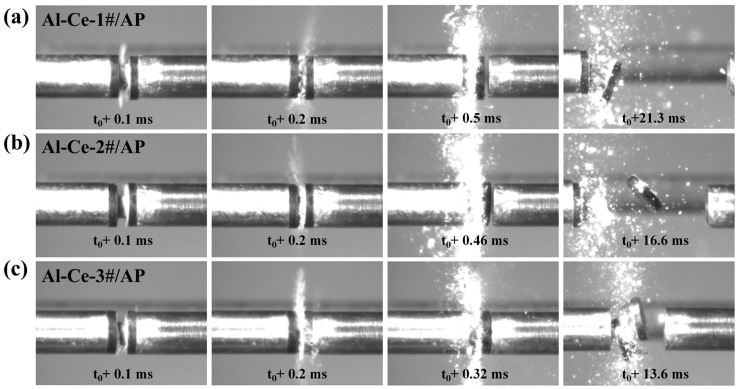
Transient ignition patterns of the Al-Ce/AP system under 19.3 m/s impact velocity. (**a**) Al-Ce-1#/AP; (**b**) Al-Ce-2#/AP; (**c**) Al-Ce-3#/AP.

When Al-Ce alloy is introduced into the Al/AP reactive material, the ignition and combustion behavior changes significantly. The Al-Ce/AP systems ignite more readily (Figure 8) and have shorter combustion durations. The combustion duration for the Al-Ce-1#/AP system from impact ignition to completion is about 21.3 ms (Figure 8a), which is 9.4% shorter than that of the Al/AP system. With a Ce content of 70%, the Al-Ce-3#/AP system has an even shorter sustained combustion time of only 13.6 ms (Figure 8c). This represents a 42% reduction compared to the Al/AP system and is accompanied by a significantly enhanced spark intensity. The introduction of Al-Ce alloy, which benefits from the high reactivity of Ce, accelerates the oxidation reaction after impact ignition, shortening the combustion cycle of the system. Moreover, higher Ce content leads to more intense reactions.

**Figure 9:**
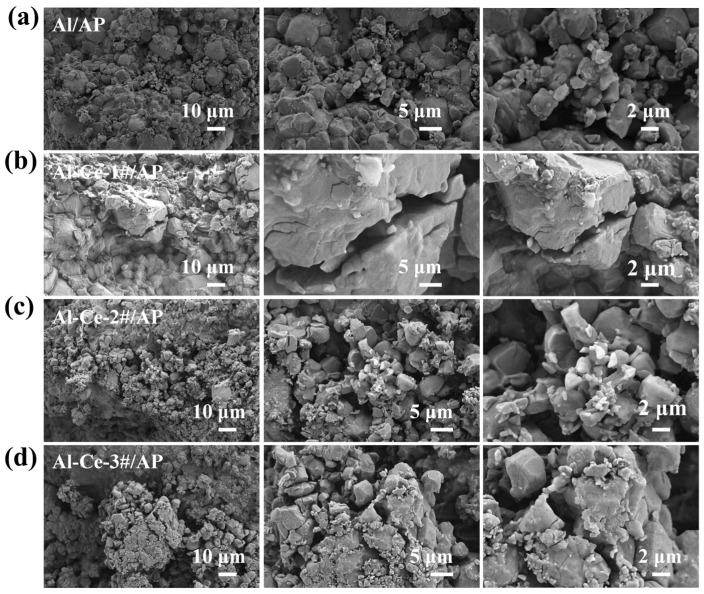
SEM images of reaction products from different AP systems under 19.3 m/s impact velocity. (**a**) Al/AP; (**b**) Al-Ce-1#/AP; (**c**) Al-Ce-2#/AP; (**d**) Al-Ce-3#/AP.

Figure 9 shows SEM images of the post-ignition residues for the four AP systems. First, it is noted that under an impact velocity of 19.3 m/s, all four AP systems can ignite upon impact but do not react completely. As seen in Figure 9a, the residue of Al/AP is largely similar in morphology to that of the original specimen (Figure 3b), with particles remaining in close contact. This indicates that heat accumulation in Al/AP after impact is poor, leading to large scale fracture with limited new reaction interfaces. In contrast, Al-Ce-3#/AP undergoes severe fragmentation upon impact, as shown in Figure 9d. The residue exhibits micro-cracks and a fragmented morphology, which provide significantly more fresh reaction interfaces.

**Figure 10:**
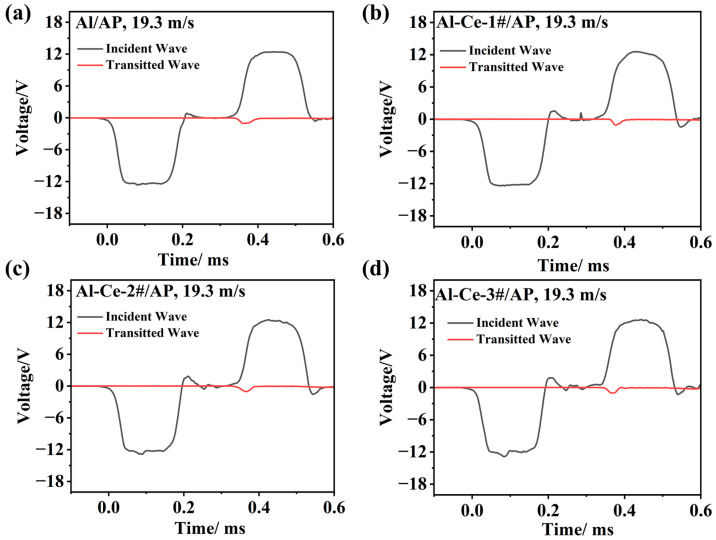
Transmitted wave and incident wave of the different metal/AP systems under 19.3 m/s impact velocity. (**a**) Al/AP; (**b**) Al-Ce-1#/AP; (**c**) Al-Ce-2#/AP; (**d**) Al-Ce-3#/AP.

Figure 10 displays the test signals for different metal/AP systems at an impact velocity of 19.3 m/s. The black solid line represents the incident stress wave pulse signal measured by the strain gauge on the incident bar, and the red dashed line represents the stress wave signal transmitted from the specimen into the transmission bar, measured by the strain gauge mounted on the transmission bar. According to the one-dimensional stress wave theory and the stress equilibrium assumption, this can be considered to reflect the pressure signal on the specimen.

**Figure 11:**
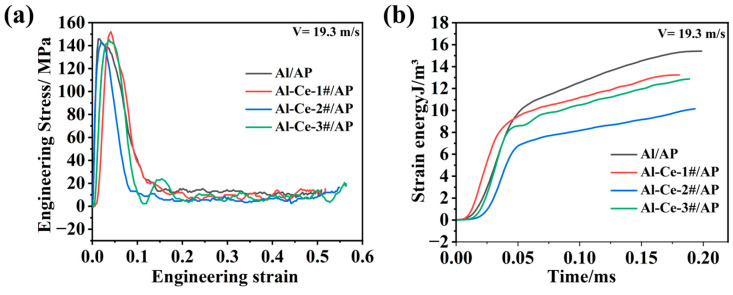
Engineering stress–strain curves (**a**) and time–strain energy curves (**b**) for different metal/AP systems under 19.3 m/s impact velocity.

Figure 11 shows the engineering stress–strain curves and strain energy versus time curves for different active metal/AP systems under an impact loading velocity of 19.3 m/s. From Figure 11a, the yield stress and ultimate strength of the four AP system specimens remain largely unchanged at 19.3 m/s. For the Al/AP system, strain energy gradually accumulates and increases with loading time, reaching a peak in the later stage of loading. Compared to Al/AP, the Al-Ce/AP systems, at the same velocity (19.3 m/s), exhibit a faster strain energy accumulation rate but achieve a lower peak strain energy density than the Al/AP system. As shown in Table 3, Al-Ce-3#/AP system fails earlier due to severe fragmentation under impact (micro-crack features in Figure 9d), preventing the material from reaching high strain energy accumulation. In contrast, Al/AP has higher ductility (large-scale fracture in Figure 9a), requiring a longer time to accumulate strain energy (peak lags in Figure 11b). It is inferred that under impact, a larger proportion of the input mechanical energy in the Al-Ce-3#/AP system is rapidly dissipated as heat and contributes to the generation of new surface area, rather than being stored as elastic strain energy. This rapid thermal dissipation facilitates the formation of localized ‘hot spots’, which, in synergy with the catalytic activity of Al_2_Ce, readily initiate exothermic chemical reactions. Figure 8c shows the shorter ignition time (13.6 ms), confirming the rapid energy release of Al-Ce-3#/AP. The reaction kinetics of the Al/AP system are slower; mechanical energy is primarily stored as strain energy and is slowly converted into thermal energy later (delayed ignition in Figure 7).

#### 3.2.2. Ignition Threshold and Mechanical Behavior Under Different Impact Velocities

**Figure 12:**
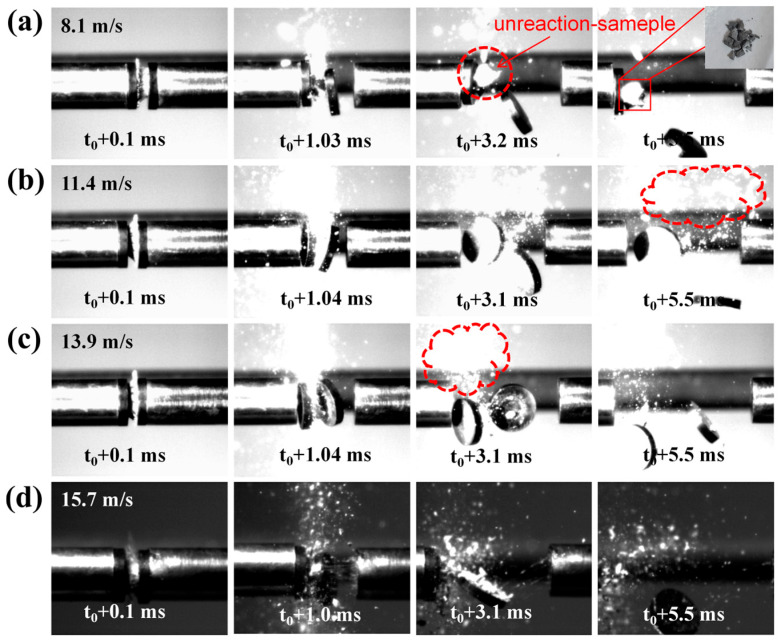
Instantaneous states of Al/AP under different impact velocity: (**a**) 8.1 m/s; (**b**) 11.4 m/s; (**c**) 13.9 m/s; (**d**) 15.7 m/s.

To further investigate the influence of Al_2_Ce on the impact ignition performance of the AP system, impact reaction experiments were conducted on the Al/AP system and the Al-Ce-3#/AP system at different impact loading velocities. Figure 12 shows the instantaneous states of the Al/AP system under different impact velocities. At an impact velocity of 8.1 m/s (loading pressure 0.1 MPa), sporadic sparks appear at 1.03 ms after impact. By 3.2 ms, portions of the impacted and fragmented specimen remain unreacted. At 5.5 ms, essentially no sparks are produced, and a large amount of unreacted sample can be observed scattering in all directions.

As the impact velocity increases from 11.4 m/s to 15.7 m/s (loading pressures 0.14 MPa to 0.3 MPa), the specimens produce no significant flame, while a large amount of unreacted sample remains. This indicates that the Al/AP system has slower reaction kinetics, a higher ignition threshold, and requires higher energy input to induce impact ignition. Based on Figure 7, the Al/AP system ignites significantly at 19.3 m/s. Therefore, the loading pressure threshold for ignition of the Al/AP system lies between 0.3 MPa and 0.4 MPa.

**Figure 13:**
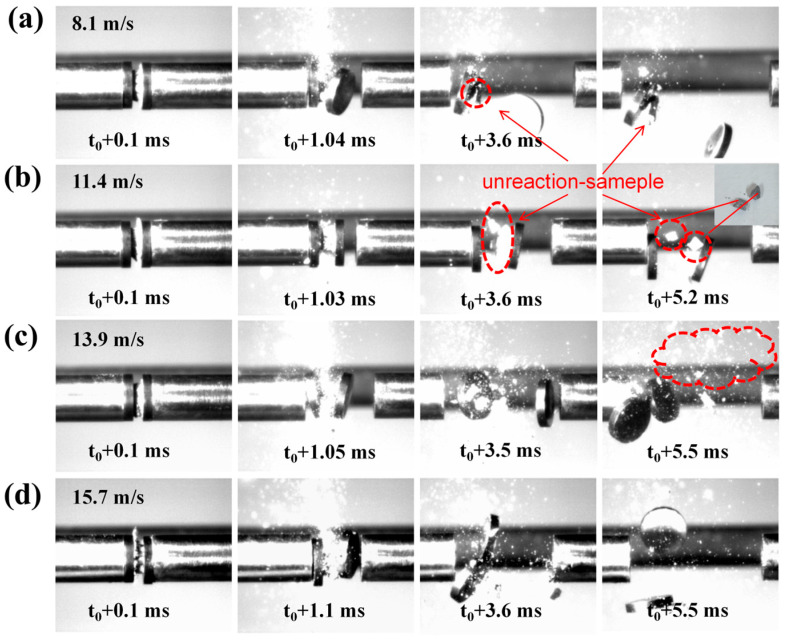
Instantaneous states of Al-Ce-3#/AP under different impact velocity: (**a**) 8.1 m/s; (**b**) 11.4 m/s; (**c**) 13.9 m/s; (**d**) 15.7 m/s.

Figure 13 shows the instantaneous states of the Al-Ce-3#/AP system under different impact pressures. At impact velocities of 8.1, 11.4, and 13.9 m/s, a portion of the unreacted sample scatters outward upon compression. This indicates that even with highly reactive metals, the system cannot accumulate sufficient energy to achieve full ignition under low energy impact. As the loading velocity further increases to 15.7 m/s, the specimen produces a noticeable flame upon impact compared to the Al/AP system. This proves that under the same loading pressure, the high reactivity of Al-Ce-3# enables the composite to react more readily, requiring a lower energy input to initiate ignition. The concentrated exothermic decomposition shown in the DSC thermal analysis results (Figure 5d) for Al-Ce-3#/AP confirms the high reactivity of Al_2_Ce. Furthermore, the more pronounced micro-crack and fragmentation features of the Al-Ce-3#/AP system after impact reaction (Figure 9d) demonstrate that Al-Ce-3#/AP more readily generates active interfaces, which are conducive to localized hot-spot formation.

**Figure 14:**
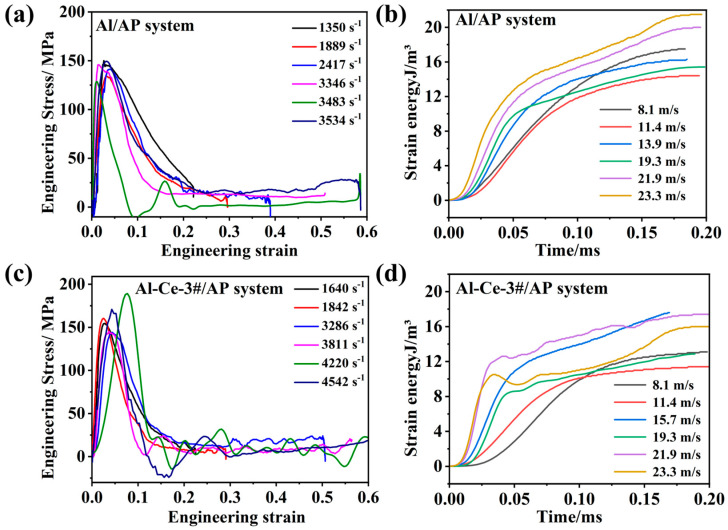
Engineering strain–stress curves for AP systems under different impact velocity: (**a**) Al/AP and (**c**) Al-Ce-3#/AP. Time–strain energy curves: (**b**) Al/AP and (**d**) Al-Ce-3#/AP.

Figure 14 presents the stress–strain curves (Figure 14a,c) and time–strain energy curves (Figure 14b,d) for the Al/AP system and the Al-Ce-3#/AP system. The curves in Figure 14a display the typical elastoplastic material characteristics of Al/AP. With increasing strain rate, the yield strength increases (strain rate strengthening effect). At higher strain rates (above 3000 s^−1^), the curves show a drop after reaching peak stress, indicating that the material undergoes damage or failure (such as cracking, particle fragmentation) under high-speed deformation. This failure behavior is the critical first step in inducing the reaction of Al/AP. The failure generates a vast new surface area and localized temperature rise (adiabatic heating), providing activation sites and initial energy for AP decomposition and Al oxidation.

Compared with Al/AP, the Al-Ce-3#/AP system generally exhibits higher yield strength and flow stress under the same impact loading velocity (Figure 14c). For example, at 21.9 m/s (~0.5 MPa), the engineering stress of Al-Ce-3#/AP approaches 190 MPa, while that of Al/AP under similar pressure is about 128.7 MPa. The specific dynamic mechanical properties are summarized in Table 4. Meanwhile, the stress drop in the Al-Ce-3#/AP system at higher strain rates appears steeper or occurs earlier.

It is known that Al_2_Ce intermetallic alloy possesses higher hardness and strength than conventional pure aluminum alloys [38]. Incorporating it into the AP matrix is equivalent to introducing stronger, harder particles into the relatively brittle AP matrix. This enhances the overall dynamic strength of the composite. Al_2_Ce particles act as reinforcing phases, bearing stress more effectively. The high reactivity of Al_2_Ce and its post-thermal reaction morphological characteristics may lead to more severe failure modes under high velocity loading (more intense particle fracture, or more significant debonding/interface cracking between particles and the AP matrix). This can be inferred from the steeper drop in the stress curves. Furthermore, the high chemical activity of Ce suggests that localized exothermic chemical reactions, such as the catalytic decomposition of AP, may be initiated earlier or more readily during deformation and failure. These reactions can directly affect local mechanical properties through mechanisms like thermal softening, thereby accelerating the overall failure process.

## 4. Conclusions

In this study, several Al-based reactive materials with different formulations were designed. The reaction characteristics of Al-Ce alloys in Al/AP systems under impact conditions were investigated by using DSC and SHPB experiments. The findings provide useful guidance for the design and application of Al-based reactive materials. The specific conclusions are as follows:

(1) The introduction of Al_2_Ce significantly catalyzes the thermal decomposition process of AP. It substantially lowers the decomposition peak temperature of AP (by up to approximately 69 °C) and promotes the merging or advancement of the low-temperature and high-temperature decomposition processes, resulting in a more concentrated and violent exothermic decomposition reaction. SEM/EDS analysis of the thermal decomposition products confirms that Al_2_Ce undergoes oxidation reactions with AP.
(2) Under SHPB dynamic loading, Al-Ce-3#/AP exhibits a fragmented, micro-cracked morphology after impact, providing numerous fresh reaction interfaces. In contrast, Al/AP primarily undergoes large-scale fracture, resulting in limited interfaces and low hot spot accumulation efficiency. At a loading velocity of 19.3 m/s, Al-Ce-3#/AP shows a faster strain energy accumulation rate but a lower peak value because mechanical energy is more efficiently dissipated as heat and fracture energy, promoting hotspot formation and the initiation of chemical reactions that release the material’s inherent chemical energy. Its higher yield strength and early failure behavior accelerate reaction initiation.
(3) Al/AP requires an impact pressure of 0.3~0.4 MPa to ignite, whereas Al-Ce-3#/AP ignites at a lower pressure of 0.3 MPa, indicating a reduced ignition threshold primarily due to the high chemical activity and catalytic effect of Al_2_Ce.

The Al_2_Ce alloy significantly enhances the impact ignition sensitivity, energy release rate, and combustion intensity of reactive materials through its high chemical activity promotion of low-temperature AP decomposition and optimization of material mechanical failure behavior. It overcomes the limitations of traditional Al-based materials in reactions under oxygen-deficient conditions or with high binder content, providing a new direction for the design of highly reactive damage materials.

## Figures and Tables

**Table 1:** Formulations of the prepared AP-based reactive material samples.

Number	Formulation	Mass Ratio (Oxygen Balance)	FKM Fraction/% (Additionally)
1#	Al/AP	28:72	5%
2#	Al-Ce-1#/AP	33:67	
3#	Al-Ce-2#/AP	37:64	
4#	Al-Ce-3#/AP	44:56	

**Table 2:** Parameters of the thermal decomposition of the four AP systems.

Component	Ratio	T/°C		Mass Loss/%
Al/AP system	28:72	334	410	69.5
Al-Ce-1#/AP system	33:67	328	357	61.0
Al-Ce-2#/AP system	37:64	329	365	50.0
Al-Ce-3#/AP system	44:56	348	357	47.0

**Table 3:** Dynamic mechanical property values and combustion times of the four AP systems.

Component	Ratio	Ultimate Strength (MPa)	Critical Failure Strain	Sustained Combustion Duration (ms)	Strain Energy (J/M^3^)
Al/AP	28:72	146.4	0.01	23.5	15.4
Al-Ce-1#/AP	33:67	151.3	0.04	21.3	13.3
Al-Ce-2#/AP	37:64	142.0	0.02	16.6	10.2
Al-Ce-3#/AP	44:56	145	0.04	13.6	13.0

**Table 4:** Dynamic mechanical property values and combustion time of Al/AP and Al-Ce-3#/AP systems.

Component	Loading Pressures (MPa)	Strain Rate (S^−1^)	Ultimate Strength (MPa)	Critical Failure Strain	Strain Energy (J/M^3^)
Al/AP	0.1	1350	145.4	0.02	17.5
	0.14	1889	133.8	0.03	14.4
	0.2	2417	141.0	0.04	16.2
	0.4	3346	146.4	0.01	15.4
	0.5	3483	128.7	0.01	20.0
	0.6	3534	149.5	0.03	21.5
Al-Ce-3#/AP	0.1	1640	154.5	0.02	13.1
	0.14	1842	160.2	0.02	11.4
	0.3	3286	143.7	0.04	17.6
	0.4	3811	145	0.04	13.0
	0.5	4220	190	0.07	17.4
	0.6	4542	170	0.04	16.0

## Data Availability

The original contributions presented in the study are included in the article, further inquiries can be directed to the corresponding authors.
